# Rapid implementation mapping to identify implementation determinants and strategies for cervical cancer control in Nigeria

**DOI:** 10.3389/fpubh.2023.1228434

**Published:** 2023-08-17

**Authors:** Ijeoma Uchenna Itanyi, Clare Viglione, Anne F. Rositch, John Olajide Olawepo, Babayemi Oluwaseun Olakunde, Akudo Ikpeazu, Uche Nwokwu, Nwamaka Lasebikan, Echezona Edozie Ezeanolue, Gregory A. Aarons

**Affiliations:** ^1^Department of Community Medicine, College of Medicine, University of Nigeria Nsukka, Enugu, Nigeria; ^2^Center for Translation and Implementation Research, University of Nigeria Nsukka, Enugu, Nigeria; ^3^UC San Diego Altman Clinical and Translational Research Institute (ACTRI) Dissemination and Implementation Science Center, La Jolla, CA, United States; ^4^Department of Epidemiology, Johns Hopkins University Bloomberg School of Public Health, Baltimore, MD, United States; ^5^Department of Health Sciences, Bouvé College of Health Sciences, Northeastern University, Boston, MA, United States; ^6^National Agency for the Control of AIDS, Abuja, Nigeria; ^7^National AIDS, Viral Hepatitis and STIs Control Programme, Federal Ministry of Health, Abuja, Nigeria; ^8^National Cancer Control Programme, Federal Ministry of Health, Abuja, Nigeria; ^9^Oncology Center, University of Nigeria Teaching Hospital, Enugu, Nigeria; ^10^HealthySunrise Foundation, Las Vegas, NV, United States; ^11^Department of Psychiatry, University of California, San Diego, La Jolla, CA, United States

**Keywords:** implementation mapping, determinants, implementation strategies, cervical cancer, Nigeria, EPIS framework

## Abstract

**Background:**

Cervical cancer constitutes a huge burden among women in Nigeria, particularly HIV-infected women. However, the provision and uptake of cervical cancer screening and treatment is limited in Nigeria. Understanding implementation determinants is essential for the effective translation of such evidence-based interventions into practice, particularly in low-resource settings. COVID-19 pandemic necessitated online collaboration making implementation mapping challenging in some ways, while providing streamlining opportunities. In this study, we describe the use of a virtual online approach for implementation mapping (steps 1–3) to identify implementation determinants, mechanisms, and strategies to implement evidence-based cervical cancer screening and treatment in existing HIV infrastructure in Nigeria.

**Methods:**

This study used a mixed methods study design with a virtual modified nominal group technique (NGT) process aligning with Implementation Mapping steps 1–3. Eleven stakeholders (six program staff and five healthcare providers and administrators) participated in a virtual NGT process which occurred in two phases. The first phase utilized online surveys, and the second phase utilized an NGT and implementation mapping process. The Exploration, Preparation, Implementation and Sustainment (EPIS) framework was used to elicit discussion around determinants and strategies from the outer context (i.e., country and regions), inner organizational context of existing HIV infrastructure, bridging factors that relate to bi-directional influences, and the health innovation to be implemented (in this case cervical cancer screening and treatment). During the NGT, the group ranked implementation barriers and voted on implementation strategies using Mentimeter.

**Results:**

Eighteen determinants to integrating cervical cancer screening and treatment into existing comprehensive HIV programs were related to human resources capacity, access to cervical cancer services, logistics management, clinic, and client-related factors. The top 3 determinants included gaps in human resources capacity, poor access to cervical cancer services, and lack of demand for services resulting from lack of awareness about the disease and servicesA set of six core implementation strategies and two enhanced implementation strategies were identified.

**Conclusions:**

Rapid Implementation Mapping is a feasible and acceptable approach for identifying and articulating implementation determinants, mechanisms, and strategies for complex healthcare interventions in LMICs.

## Introduction

Cervical cancer is a challenging chronic disease affecting millions of women in sub-Saharan Africa. Cervical cancer is the second most common cancer affecting women in Nigeria, accounts for the highest number of deaths from cancers, and is more prevalent in HIV-infected women and occurs at a younger median age than in HIV-negative women (1, 2). The provision and uptake of cervical cancer screening and treatment is limited in Nigeria (3, 4). There are key gaps in understanding implementation determinants that impact implementation of cervical cancer screening and treatment in HIV clinics across Nigeria.

Core to dissemination and implementation science is the identification of implementation determinants and mechanisms (i.e., impediments or facilitators to successful implementation of evidence-based innovations) along with the articulation and testing of strategies to tackle identified determinants (5). Determinants and mechanisms are not always obvious, and their identification requires partnership and engagement with community members, practitioners, and on-the-ground implementers to harvest the practical wisdom and knowledge to uncover and contextualize them. For the purposes of this project, we use the term determinants to represent determinants and mechanisms while understanding that some determinants may act as mechanisms. Implementation determinants are myriad and exist at any level of the socio-ecological spectrum, from the outer context (e.g., policies, social determinants of health) to the inner context (e.g., provider organizations), and it is essential to delineate determinants and address them to ensure the successful implementation, adoption and sustainment of evidence-based interventions (6).

Implementation mapping is a method aimed at helping to identify determinants, mechanisms, and strategies relevant for implementing evidence-based interventions in specific contexts. A key limitation of implementation mapping is that it can be time and resource intensive and fairly onerous to participants, requiring multiple focus group meetings spanning weeks or months, multiple iterations to the protocol with built-in time for discussion, and rounds of testing and debriefing (7–9). While engaging community partners and collaborators, it is important to be respectful of time and minimize burden (10, 11). Furthermore, the COVID-19 pandemic has accelerated the need for research and implementation science such that it is important to adapt methods to improve the efficiency of implementation methods and innovation across healthcare settings. The pandemic has also required teams to rapidly shift to virtual spaces and often rely fully on virtual collaboration, even in low- and middle-income countries (LMICs). Although online platforms allow multiple users to synchronously connect with built-in mechanisms for chatting, facilitated group conversations online are sometimes impeded by technical difficulties, voice interruptions, and predictable environmental distractions (12–14).

Due to the necessity of virtual collaboration since March 2020, and the potential benefits of implementation mapping for large-scale geographically dispersed project, implementation mapping has needed to be modified for virtual platforms and for different service settings (15). In fact, there is nothing inherent in implementation mapping that requires face-to-face interaction. In this study, we utilized an online format of nominal group technique (NGT) combined with Rapid Implementation Mapping process (i.e., steps 1–3) to identify determinants, mechanisms, and strategies to implement and sustain cervical cancer screening and treatment uptake in HIV clinical settings in all six regions of Nigeria (16) for a National Cancer Institute grant application, now funded. In this paper, we describe the use of an adapted protocol of implementation mapping to rapidly identify and contextualize determinants to cervical cancer screening and treatment, map determinants to implementation strategies, and define a set of core and enhanced strategies for cervical cancer control implementation in Nigeria.

## Materials and methods

### Study context

This study was designed and conducted by a core team of researchers from the University of Nigeria Nsukka (UNN), Northeastern University, Johns Hopkins University, and the University of California San Diego. This study is part of a research collaboration among the universities and six major HIV implementing partners in Nigeria, who are also members of the Nigeria Implementation Science Alliance. In 2021, the Nigeria Implementation Science Alliance established 21 Model Innovation and Research Centers for multi-center clinical trials and implementation research. The process of establishment of these model centers has been reported elsewhere (17). The HIV prevention leads from the six implementing partners collaborated with the research team to conduct a needs assessment for integrating cervical cancer screening and treatment into the existing comprehensive HIV program in Nigeria.

### Study design, participants, and data collection

This was a modified version of NGT with group brainstorming and ranking. We utilized the Exploration, Preparation, Implementation, Sustainment framework to guide and contextualize our activities and goals (18, 19). EPIS is both a process and determinant framework (i.e., dynamic framework) that is useful for collaborators in considering determinants and mechanisms across the four phases—Exploration, Preparation, Implementation and Sustainment. EPIS is useful in study design and execution in order to identify determinants and mechanisms, and related measures and activities that may occur during all four EPIS phases (20). The main EPIS determinants constructs included outer system context, inner organizational context, bridging factors that represent bi-directional linkages and relationships between outer and inner contexts, innovation characteristics including engagement of intervention developers, and interconnections and linkages within and across contexts and constructs. We describe our activities in the Exploration phase of EPIS to identify the determinants and select implementation strategies for cervical cancer control in Nigeria.

We invited eleven participants (nine program staff and two healthcare providers) to participate in an implementation mapping process that occurred in two phases. The first phase utilized an online survey, while the second phase utilized a virtual NGT. Participants for the online survey included the HIV prevention leads who were the program leads for Prevention of Mother-to-Child transmission of HIV program and comprised lead of each of the six major implementing partners in Nigeria. Participants for the NGT were five of the six HIV prevention leads described above and five health facility staff (two healthcare providers and three program staff). The health facility staff were purposively selected from health facilities supported by these major implementing partners based on their engagement and responsiveness with the NISA-MIRCs team.

### Description of implementation mapping

Implementation mapping is a systematic process for developing strategies to improve the adoption, implementation, and sustainment of evidence-based interventions in real-world settings. Implementation mapping involves five activities: (i) conduct an implementation needs assessment and identify implementers; (ii) identify implementation outcomes, determinants, and create matrices of change objectives; (iii) choose theoretical methods (mechanisms of change) and select or design implementation strategies; (iv) produce implementation protocols and materials; and (v) evaluate implementation outcomes (16).

### Rapid implementation mapping process

**Figure 1:**
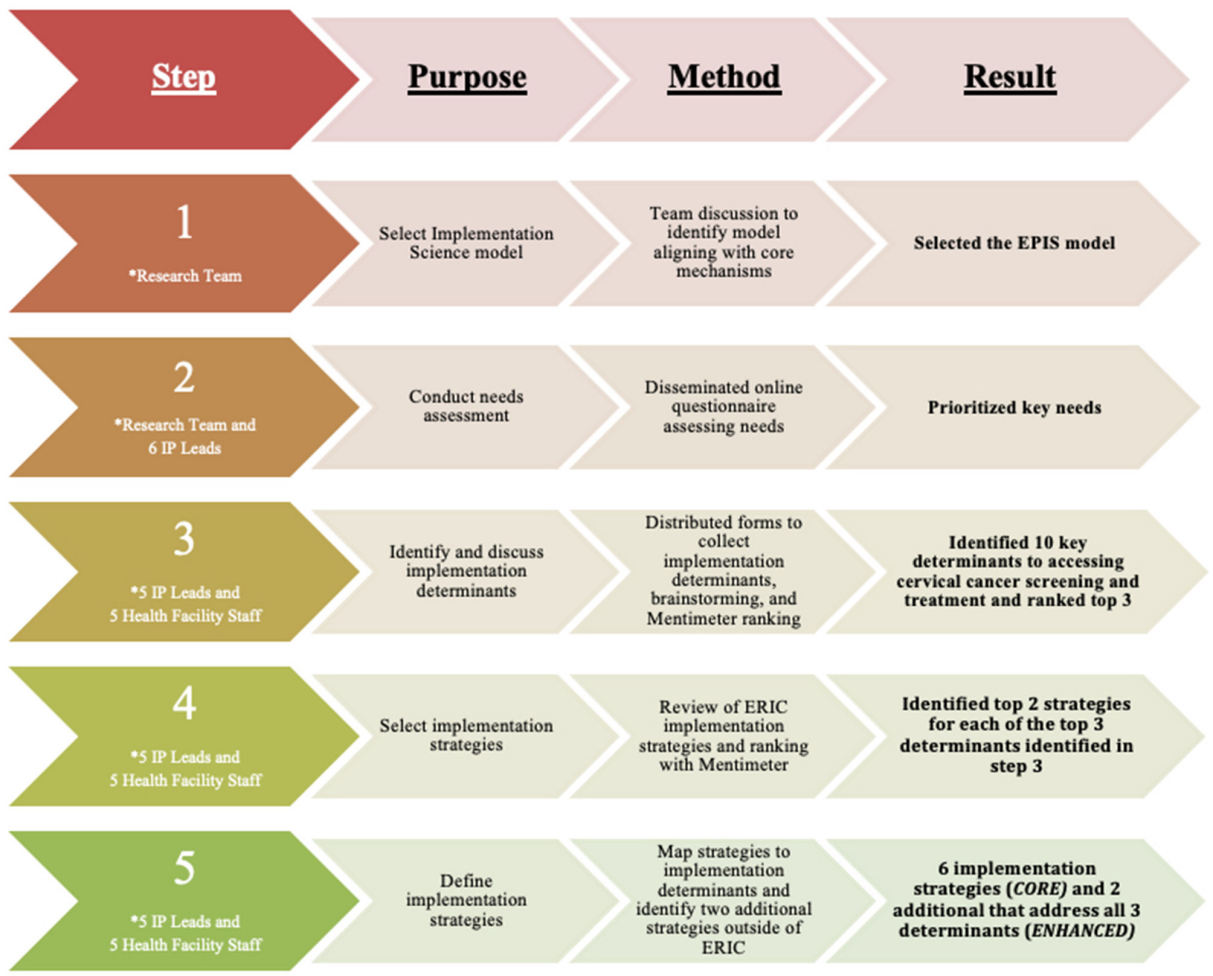
The rapid implementation mapping protocol. *Participants in each step of the protocol.

This rapid implementation mapping process occurred in two phases. Figure 1 summarizes the process.

#### Phase 1: online survey

The research team approached the six HIV prevention leads by email and invited them to participate in an online survey. The team introduced the goal of the survey (to identify determinants, mechanisms, and potential implementation strategies for integrating cervical cancer screening and treatment into existing comprehensive HIV treatment programs), and shared the refined compilation of implementation strategies of the Expert Recommendations of Implementing Change (ERIC) project journal article (21) with them. These partners were asked to identify three anticipated critical determinants (barriers) to integrating cervical cancer screening and treatment into existing comprehensive HIV treatment programs, and select five potential implementation strategies from the ERIC taxonomy to address their three most critical identified determinants (21). The survey included the following two questions: “(1) What are three critical anticipated barriers to integrating cervical cancer screening into the existing HIV program;” and “(2) List five potential implementation strategies for addressing your three identified determinants in Question 1 above (Please choose from the attached journal article).” They completed and submitted the survey after 1 week.

#### Phase 2: NGT

In the second phase, the six HIV prevention leads and five health facility staff, one from each of five implementing partner-supported clinics (total of 11 partners) were invited for a brainstorming session and virtual NGT on Zoom. One of the HIV prevention leads could not attend the Zoom session, so we had 10 participants. The health facility staff received phone credits to access internet data for the Zoom meeting. In preparation for the NGT, the research team met to develop and refine a seven-step process for the virtual NGT. Ten partners participated in the Zoom session which followed a seven-step process building on the results of the determinants and implementation strategies survey. The virtual NGT was led by three members of the research team who have facilitated NGT in the past (22) with IUI leading Steps 1 to 5, and BOO and EEE leading Steps 6 and 7.

**Step 1:** The research team collated all the 18 identified determinants and selected implementation strategies from each HIV prevention lead (n=6). The research team then grouped these 18 determinants into 10 based on repetitions and their similarities in preparation for a rapid version of NGT.
**Step 2:** The participants first reviewed and agreed on the initial grouping of the 18 determinants into 10 determinants by the research team. Each of the 10 participants was then asked to define and explain his/her identified implementation determinant using the “name it, define it, and operationalize it” approach (23). Consistent with NGT, this step used a focus group discussion approach where all 10 participants were given the opportunity to define the 10 identified determinants. During these discussions, two additional determinants emerged. After all the 10 initial and two additional determinants were defined, the participants grouped similar determinants together and reached a consensus on a final grouping and naming of 10 determinants.
**Step 3:** The participants were asked to select their top implementation determinants based on importance (if addressed, will help overcome the gaps in cervical cancer prevention and control—screening, onsite treatment and referral among HIV-infected women) and feasibility of addressing them. We used Mentimeter, an online polling tool, to allow the group members to confidentially rank each determinant. The aggregated group-level data were then used to identify the collective three most important and feasible implementation determinants.
**Step 4:** For each of the top three implementation determinants, each participant was asked to match and rank the top potential implementation strategy from the initially selected ERIC implementation strategies during the survey. The instructions for this activity were to use perceived importance and feasibility as criteria for ranking the top implementation strategies. Mentimeter was used for the ranking and selection of the top two implementation strategies to address each of the top three determinants identified in Step 3. When there was a tie in the ranking, a tie breaker was applied by having participants again make ratings in Mentimeter. There were ties in the ranking of the top two implementation strategies for the second and third implementation determinants, and these were resolved with tie breakers.
**Step 5:** The group (participants and research team) defined the six selected implementation strategies in Step 4 as the Core implementation strategies selected from the ERIC set of strategies to address the group's top three determinants to integrating cervical cancer screening and treatment into existing comprehensive HIV programs.
**Step 6:** The group proposed and discussed additional implementation strategies outside the ERIC project's compilation of implementation strategies, relevant to Nigeria and other LMICs and can be culturally tailored to the country and region. This step was important because not all potential strategies are represented in existing listings. The criteria for proposing these additional implementation strategies were based on: (1) importance; (2) feasibility; (3) can address >1 implementation determinant in the three main stages of the cervical cancer identification and treatment cascade (screening, onsite treatment, and referral); and (4) can be implemented across all the 12 implementation sites. During the discussion, all participants were encouraged to contribute and the team agreed on a set of six additional implementation strategies.
**Step 7:** The participants ranked their top additional implementation strategy based on the four criteria defined in Step 6, using Mentimeter. There was a tie between the top second and third additional implementation strategies and by consensus, the group agreed to use a blended strategy for this tie. At the end of this step, the group defined the top three ranked additional implementation strategies as the enhanced set of implementation strategies to address the group's top three determinants to integrating cervical cancer screening and treatment into existing comprehensive HIV programs. This was consistent with the goal to identify a core multifaceted implementation strategy and a core+ multifaceted strategy that could be tested in a randomized comparative effectiveness implementation study.

## Results

### Characteristics of participants

The HIV prevention leads included four physicians and two nurses. All but one had a terminal degree (MD or PhD), and all had at least 14 years' experience working in the health sector. The health facility staff included one physician gynecologist, one registered nurse, and three monitoring and evaluation officers. Two of the monitoring and evaluation officers had a master's degree while the third has a bachelor's degree. All but one had at least 5 years' experience working in the health sector.

### Phase 1

The six HIV prevention leads identified 18 determinants (Supplementary Table 1) to integrating cervical cancer screening and treatment into existing comprehensive HIV programs. These determinants were related to human resources capacity, access to cervical cancer services, logistics management, clinic, and client-related factors. These determinants were grouped into 10 determinants by the research team based on repetition and similarities as described in the methods (Supplementary Table 2). Each HIV prevention lead also identified three to five implementation strategies from the ERIC strategies for each identified determinant resulting in a total of 9–15 implementation strategies for each HIV prevention lead.

### Phase 2

At the end of Step 2, the five HIV prevention leads and five healthcare providers (10 participants in total) named, defined, and operationalized a final set of 10 determinants to integrating cervical cancer screening and treatment into existing HIV programs (Supplementary Table 3). During this step, the participants merged initial determinants 3 (i.e., lack of demand for services) and 9 (i.e., education about disease and services). Similarly, determinant 10 (i.e., access to patients) was merged with determinant 2 (i.e., poor access to cervical cancer services with insufficient treatment sites). The two additional determinants which emerged were stigma, and lack of adoption of guidelines at implementation sites/clinics. After ranking, the top three determinants selected by the participants included 1) gap in human resources capacity, 2) poor access to cervical cancer services with insufficient treatment sites/access to patients, and 3) lack of demand for services resulting from lack of awareness about the disease and services.

**Figure 2:**
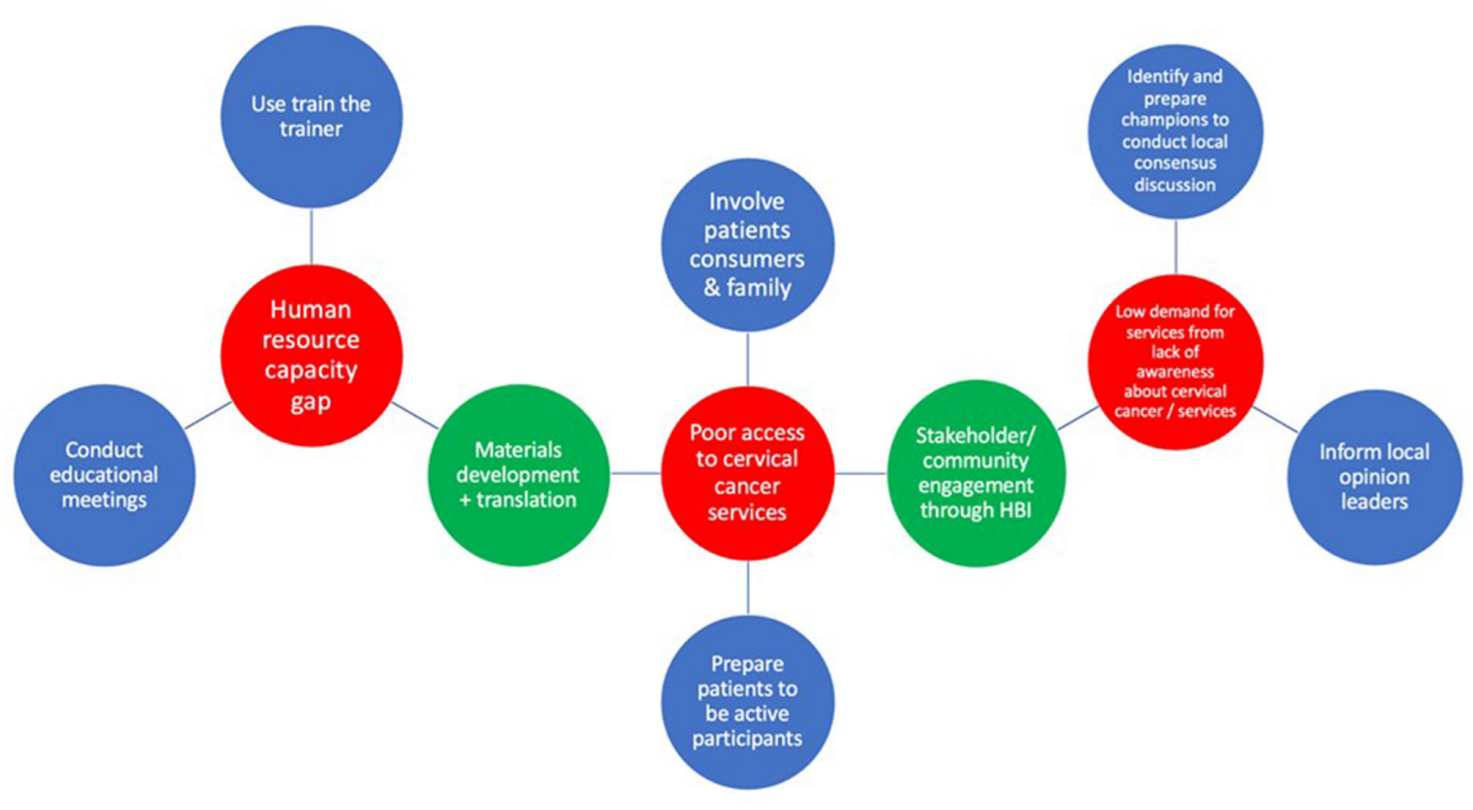
Matching core and enhanced implementation strategies to implementation determinants. Key: determinants (red), core implementation strategies (blue), enhanced implementation strategies (green).

Three implementation strategies were ranked for the determinant “gaps in human resources capacity” and there were no ties. For the determinant of “poor access to cervical cancer services with insufficient treatment sites/access to patients,” five implementation strategies were ranked and there was a tie between prepare patients/consumers to be active participants and alter patient/consumer fees. After breaking the tie, prepare patients/consumers to be active participants ranked second with seven votes. Four implementation strategies were ranked for “lack of demand for services resulting from lack of awareness about the disease and services.” There was a tie between identify and prepare champions and conduct local consensus discussions. The latter received five votes while the former received four votes during the tie breaker voting. One of the participants (an HIV prevention lead) could not vote for the tie breaker because of poor internet connectivity. Following the inconclusive outcome of the votes, the group agreed to select conduct local consensus discussions (blended with identify and prepare champions) as the second implementation strategy for “poor access to cervical cancer services with insufficient treatment sites/access to patients.” At the end of Step 5, the participants had selected and defined a set of six core implementation strategies to address the top three potential determinants to cervical cancer integration (Figure 2).

Six additional implementation strategies, not originating from the ERIC set of strategies were proposed. The research team had suggested the Healthy Beginning Initiative, popularly known as “Baby Shower” (24) and the use of mobile health smartcard technology (both locally developed, tested, and implemented strategies in prior work focused on increasing access to the delivery of health interventions and follow up) (25). The participants proposed the remaining four implementation strategies. During ranking, the Healthy Beginning Initiative/Baby Shower and stakeholder engagement tied for the second place and there was a consensus by the group to use both strategies as a blended implementation strategy (Stakeholder [community] engagement through the Healthy Beginning Initiative). Figure 2 shows the final set of enhanced implementation strategies defined by the group.

## Discussion

We outline a rapid implementation mapping (steps 1–3) protocol to identify implementation determinants, and strategies to implement evidence-based cervical cancer screening and treatment in the existing HIV programs in Nigeria. We demonstrate the feasibility and acceptability of implementation mapping with modified NGT to uncover determinants to, and strategies for implementation of, cervical cancer screening in Nigerian clinics. Our experiences underscore that implementation mapping can be an efficient and pragmatic overarching framework when combined with NGT for consensus building to select determinants and strategies.

Implementation Mapping, and the Intervention Mapping protocol from which Implementation Mapping was derived, are traditionally time and resource intensive requiring multiple meetings across weeks or months to articulate implementation plans (9). For engaging clinicians and community partners, it is critical to respect time, support meeting access, and minimize burden (10, 11). Other consensus-building techniques and approaches including user-centered design protocols, Delphi techniques, or concept mapping which can be time-consuming potentially causing protracted research delays and slowing public health impact (26). Moreover, the pandemic has accelerated the pace of research and highlighted the need to quickly optimize interventions for implementation and scale from the outset. In this rapid version of implementation mapping (steps 1–3), consisting of an electronic survey (20 mins) and a facilitated Zoom meeting (165 mins), it took ~3 h and 5 mins in total to identify a set of Core and Enhanced Implementation Strategies within 5 days. This is contrasted with the aforementioned consensus building approaches like concept mapping which can be more time-consuming and not as agile and engaging when done remotely (27). The virtual platform and Mentimeter voting tool were instrumental to accelerate the process of implementation mapping and NGT. Specifically, Mentimeter voting happened synchronously within seconds through a password-protected website shared in the Zoom chat which was accessible on any web-enabled device (e.g., smart phone, tablet, or computer). The availability of internet network facilitated this virtual implementation mapping process. Also, the provision of data to the health facility staff helped overcome the limitation of inadequate data for the 2 h 45 mins Zoom meeting. However, poor network in some locations resulted in interruptions for some participants who were disconnected from the Zoom meeting occasionally and they had to rejoin the meeting.

EPIS served as a helpful framework to stimulate discussions around potential determinants and strategies from the outer system (i.e., country and regions) context, inner organizational context of existing HIV infrastructure, bridging factors that relate to bi-directional influences, and the health innovation to be implemented (in this case cervical cancer screening and treatment). The NGT participants engaged in discussions of how EPIS applied to the proposed project and need to consider all of the EPIS phases and factors. Of the 10 determinants of cervical cancer screening for HIV-infected women in Nigeria, NGT participants selected determinants spanning different levels of EPIS 1) lack of human resources (outer system), 2) poor access to cervical cancer screening (bridging factor), and 3) low awareness/low demand for services (inner context, individual level).

A possible criticism of rapid implementation mapping may include the minimization of group discussion in favor of rapid consensus building using ranking and voting. However, despite using a tightly structured agenda with rounds of voting, there were also several opportunities for open conversation using a “round robin” focus group discussion style, allowing participants to articulate and contextualize determinants to better understand which determinants and strategies might be most impactful. Words like “meaningful,” “feasible,” “appropriate,” and “important” were used by participants to discuss strategies which naturally encouraged the group to clarify priorities and think through the potential impact of selecting specific determinants. Although consensus was solidified quickly through voting, one could argue that through the rapid implementation mapping and NGT, all voices are elevated, and hierarchies are flattened. In fact, NGT has been described as a technique for effective group process in community-based participatory research partnerships because it allows equitable participation and open communication (28).

Strengths of this rapid implementation mapping protocol include the multi-step and systematic process for pre-meeting data collection, anonymous in-person voting, and facilitated discussion. Additionally, the use of multiple methods to triangulate data collection through survey, focus group discussion, and voting is an important strength. Lastly, this rapid implementation mapping protocol has the potential to promote health equity by involving communities in identifying implementation determinants that cause health disparities and selecting context-specific implementation strategies that can lead to successful implementation of evidence-based interventions and improved health outcomes. Limitations include the single case study which may limit application and generalizability to other research teams and settings. In the current context, research team members spanned Nigeria and the United States, and most team members and stakeholders had previously worked together. Inclusion of global colleagues can be challenging when there is poor team dynamics and may be more time consuming and costly in a non-virtual environment.

## Conclusion

We outline the feasible and efficient use of a virtual protocol of Rapid Implementation Mapping to identify implementation determinants and strategies to implement evidence-based cervical cancer screening and treatment in existing HIV treatment programs in Nigeria. As COVID-19 has necessitated online collaborations and approaches in dissemination and implementation science, modified virtual implementation mapping can help keep up with equitable implementation efforts in low-income settings.

## Data availability statement

The original contributions presented in the study are included in the article/Supplementary material, further inquiries can be directed to the corresponding author.

## Ethics statement

This was a quality improvement project involving members of the Nigeria Implementation Science Alliance hence ethics approval was not required according to the national ethics guidelines. The study was conducted in accordance with the local legislation and institutional requirements. According to national ethics guidelines for quality improvement projects, written informed consent was not required. However, we obtained verbal and implied consent through participants' responses to emails and acceptance of invitations to the nominal group technique. No potentially identifiable images or data are presented in this study.

## Author contributions

IUI, CV, JOO, BOO, EEE, and GAA conducted the implementation mapping process. IUI and CV wrote the first draft of the manuscript while all authors revised the manuscript for important intellectual content. All authors read and approved the final draft.
