# Supplementary material for: Rapid implementation mapping to identify implementation determinants and strategies for cervical cancer control in Nigeria
Source: Front Public Health. 2023 Aug 17;11:1228434. doi: 10.3389/fpubh.2023.1228434 (PMC10469679; doi:10.3389/fpubh.2023.1228434)
Supplement: Supplementary file 1 [file Table_1.docx]

**Supplementary Table 1: Determinants to integrating cervical cancer services into existing HIV programs, identified by implementing partners during online survey**

| **Implementing Partner** | **Determinants** |
| --- | --- |
| IP 1 | Human resources gap and capacity |
|  | Poor access and coverage of cervical cancer screening services |
|  | Lack of demand for services by locals |
| IP 2 | Inability of women to pay for further evaluation (e.g., pap smear) after positive VIA result |
|  | Insufficient treatment centers across the supported states |
|  | Occasional stockout of screening commodities |
| IP 3 | Hospital barriers (Long client waiting time, distance to the facility, cost of screening /treatment) |
|  | Psychological barriers (fear of result, spouse-related issues, treatment options and fear of not conceiving after screening) |
|  | Educational barriers (knowledge gap, communication gap, literacy level) |
| IP 4 | Inadequate human resources to implement routine screening program |
|  | Poor coverage of PLHIV for cervical cancer screening (multi- months drugs dispensing) |
|  | Inadequate treatment centers |
| IP 5 | Lack of infrastructure (few facilities had any form of expertise in screening prior to service delivery) |
|  | Quality of care (not all facilities had treatment modalities for precancerous lesions and fewer can manage suspected cancer cases, false positives and false negatives as VIA is subjective) |
|  | Service access (clients living in hard-to-reach areas, industrial unrest causing service interruption) |
| IP 6 | Stock out of consumables (e.g., disposable speculums for screening) |
|  | Insufficient thermal ablation machine / Insufficient manpower |
|  | Limited number of trained staff for cervical cancer |
