# Supplementary material for: Rapid implementation mapping to identify implementation determinants and strategies for cervical cancer control in Nigeria
Source: Front Public Health. 2023 Aug 17;11:1228434. doi: 10.3389/fpubh.2023.1228434 (PMC10469679; doi:10.3389/fpubh.2023.1228434)
Supplement: Supplementary file 2 [file Table_2.docx]

**Supplementary Table 2: Initial grouping of determinants to integrating cervical cancer services into HIV programs**

| **S/N** | **Determinants** |
| --- | --- |
| 1 | Gap in human resources capacity |
| 2 | Poor access to cervical cancer services with insufficient treatment sites |
| 3 | Lack of demand for services |
| 4 | Inability to pay for services |
| 5 | Stockout of materials |
| 6 | Facility determinants like long waiting times |
| 7 | Personal psychological determinants like fear of results |
| 8 | Cultural determinants like role of male partner |
| 9 | Awareness/education determinants about disease and services |
| 10 | Access to patients (e.g., multi-month dispensing) |
