# Supplementary material for: Rapid implementation mapping to identify implementation determinants and strategies for cervical cancer control in Nigeria
Source: Front Public Health. 2023 Aug 17;11:1228434. doi: 10.3389/fpubh.2023.1228434 (PMC10469679; doi:10.3389/fpubh.2023.1228434)
Supplement: Supplementary file 3 [file Table_3.docx]

**Supplementary Table 3: Final compilation of 10 determinants to integrating cervical cancer services into HIV programs**

| **Implementation Determinants** | |
| --- | --- |
| Gap in human resources capacity | Stock-out of materials |
| Poor access to cervical cancer services with insufficient treatment sites | Facility barriers like long waiting times and limited space |
| Lack of demand for services resulting from lack of awareness about the disease and services | Personal psychological barriers like fear of results |
| Cultural barriers like role of male partner | Stigma |
| Inability to pay for services | Lack of adoption of guidelines at implementation sites/clinics |
